# Machine learning-driven prediction of heavy metal pollution for aquatic health surveillance and disease control

**DOI:** 10.1016/j.isci.2026.116549

**Published:** 2026-07-07

**Authors:** Md. Abdullah Al Mamun Hridoy, Pakorn Ditthakit, Akib Hosen, Mohd. Saifur Rahman, Minhaj Uddin, Kamrul Hasan, Yeaman Hossain, Chiara Bordin, Leonardo Goliatt, Md. Abdullah Al Mamun, Paolo Pastorino

**Affiliations:** 1 Faculty of Fisheries, Sylhet Agricultural University, Sylhet 3100, Bangladesh; 2 Center of Excellence in Sustainable Disaster Management, School of Engineering and Technology, Walailak University, Nakhon Si Thammarat 80161, Thailand; 3 Department of Environmental Science, Bangladesh Agricultural University, Mymensingh 2202, Bangladesh; 4 Department of Agricultural Construction and Environmental Engineering, Sylhet Agricultural University, Sylhet 3100, Bangladesh; 5 Department of Agricultural and Biological Engineering, Mississippi State University, 130 Creelman Street, Mississippi State, MS 39762, USA; 6 Faculty of Agricultural Economics and Business Studies, Sylhet Agricultural University, Sylhet 3100, Bangladesh; 7 Department of Computer Science, UiT The Arctic University of Norway, 6050, Langnes, 9037 Tromsø, Norway; 8 Department of Computational and Applied Mechanics, Federal University of Juiz de Fora, Juiz de Fora 36036-900, Brazil; 9 Department of Fish Health Management, Laboratory of Fish Diseases Diagnosis and Pharmacology, Faculty of Fisheries, Sylhet Agricultural University, Sylhet, Bangladesh; 10 Faculty of Veterinary Medicine, Universitas Brawijaya, Malang, East Java, 65151, Indonesia; 11 Experimental Zooprophylactic Institute of Piedmont, Liguria and Aosta Valley, Via Bologna 148, 10154 Turin, Italy

**Keywords:** heavy metal, water quality, early warning systems, prediction model, machine learning

## Abstract

Heavy metal (HM) accumulation in lake sediments poses ecological risks and may increase aquatic organism vulnerability to disease, requiring cost-effective monitoring tools. This study developed an interpretable multi-output machine learning (ML) framework to predict sediment HMs (As, Cd, Cu, Hg, Ni, Pb, and Zn) in Swan Lake, using field variables (pH, dissolved oxygen, and temperature), sediment texture (median grain size), and aluminum content (Al%). Models with nested preprocessing and cross-validation achieved strong performance (R^2^ = 0.824–0.997). Optimal algorithms varied by metal, while random forest showed the most consistent results, balancing accuracy, low error, and robustness to outliers. Predicted concentrations were integrated with ecological risk indices (Igeo, PLI, and Hakanson RI), indicating low pollution with localized Zn hotspots. Explainability analysis identified pH and Al% as key drivers, supporting ML-based screening for aquatic monitoring and early warning systems.

## Highlights

• Multi-output machine learning predicts sediment heavy metals for aquatic health monitoring
• Random forest provides robust performance across multiple heavy metals
• Model predictions align with standard ecological risk indices
• Explainability identifies pH and Al% as dominant geochemical drivers

## Graphical abstract

**Figure:**
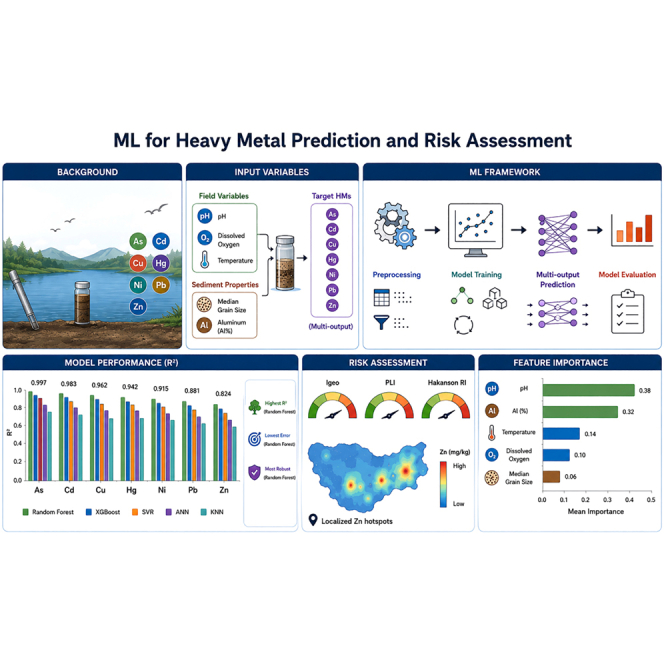

## Teaser

Environmental health; environmental toxicology; machine learning

## Introduction

Heavy metal (HM) contamination in aquatic ecosystems has become a critical environmental issue driven by rapid industrialization, urban expansion, agricultural intensification, and climate-driven hydrological variability.^1^^,^^2^ Metals such as arsenic (As), cadmium (Cd), copper (Cu), mercury (Hg), nickel (Ni), lead (Pb), and zinc (Zn) are of particular concern because of their persistence, non-biodegradable nature, and strong tendency to bioaccumulate and biomagnify through aquatic food webs.^3^^,^^4^ Once introduced into water bodies, these elements can alter sediment-water exchange processes, disrupt microbial and biogeochemical cycling, and pose long-term risks to aquatic biodiversity and human health.^5^ Consequently, reliable tools for monitoring and predicting HM concentrations are essential for ecosystem protection and sustainable water-resource management.^6^^,^^7^^,^^8^

Conventional assessments of HM contamination in aquatic environments have predominantly relied on laboratory-intensive chemical analyses combined with sediment quality indices such as the geoaccumulation index (I_geo), enrichment factor (EF), pollution load index (PLI), and potential ecological risk index (RI).^9^^,^^10^ These indices provide standardized means for quantifying the contamination intensity and ecological risk and remain widely adopted in limnological and environmental geochemistry research.^11^ However, reliance on spatially sparse sampling and time-consuming laboratory workflows often limits temporal and spatial resolution, constraining timely detection of contamination hotspots and proactive risk management.^12^ Therefore, scalable predictive approaches that leverage readily measurable environmental variables are increasingly needed to complement traditional monitoring.^13^

Recent advances in environmental informatics have introduced machine learning (ML) as a promising approach for predicting water quality parameters and contaminant behavior, including HM concentrations.^14^^,^^15^ Common ML models, random forest (RF), support vector machine (SVM), artificial neural networks (ANNs), and gradient boosting frameworks such as XGBoost and LightGBM, have demonstrated strong predictive performance in complex aquatic datasets by capturing non-linear relationships and interaction effects among predictors.^15^^,^^16^ In both water-column and sediment contexts, ML models have been used to estimate metal concentrations from physicochemical and geochemical proxies, including pH, dissolved oxygen (DO), temperature, sediment grain size (φ) distribution, and major element oxides such as Al and Fe that influence sorption and partitioning processes.^17^^,^^18^ Mechanistically, pH governs metal solubility and adsorption-desorption kinetics, DO modulates redox conditions relevant to redox-sensitive elements (e.g., As and Hg), temperature affects reaction kinetics and diffusion rates, and finer grain sizes generally increase surface area and binding capacity, while aluminum/iron oxides act as key sorbents in sediments.^18^^,^^19^

Despite the expanding adoption of ML in aquatic contamination assessment, several gaps remain. First, many studies emphasize single-output prediction (one metal at a time), whereas environmental management often requires an integrated assessment across multiple metals under shared drivers and coupled geochemical controls; consequently, multi-output prediction frameworks remain comparatively underrepresented in lacustrine sediment systems.^20^ Second, many ML studies prioritize predictive accuracy while providing limited interpretability, reducing the ability to relate model behavior to established sediment geochemistry and to translate findings into mechanistic insight.^21^ Model explainability methods, such as SHAP (Shapley Additive Explanations), permutation importance, and partial dependence analysis, have improved interpretability in water quality modeling and can reveal dominant predictors controlling HM variability.^22^^,^^23^ Nevertheless, explicit integration of explainability outputs with classical contamination and ecological risk indices (e.g., I_geo, EF/PLI, and RI) remains limited, sustaining a disconnect between data-driven predictions and decision-relevant risk frameworks.^24^^,^^25^

The rationale for target metal selection and scope is grounded in both environmental relevance and data completeness. The metals As, Cd, Cu, Hg, Ni, Pb, and Zn are widely prioritized in sediment quality monitoring because of their persistence, toxicity, and potential for bioaccumulation, and they are routinely included in the contamination and ecological risk assessment frameworks. In addition, these seven metals were consistently available with sufficient completeness in the referenced Swan Lake dataset, enabling a reproducible multi-output modeling benchmark. The present work evaluates the predictive performance and interpretability within the Swan Lake domain. To reduce site-specific optimism, robustness was further assessed using spatially blocked validation; however, full external validation across independent lake/lagoon systems with distinct hydrology and source regimes remains necessary and is identified as a key next step.^26^

Against this background, the study develops and validates an integrated, multi-output ML framework to predict HM concentrations and associated ecological risk indices in lake sediments. The specific objectives are to (1) model and predict the concentrations of As, Cd, Cu, Hg, Ni, Pb, and Zn, using readily obtainable physicochemical and geochemical parameters (pH, DO, temperature, φ, and aluminum content [Al%]); (2) compute standard sediment contamination and risk indices (I_geo, EF/PLI, and RI) from both measured and ML-predicted values; and (3) interpret model behavior using explainability approaches to identify dominant geochemical drivers of metal accumulation. By coupling multi-metal prediction with explainability and established risk indices, the proposed framework provides a cost-effective and scalable complement to conventional monitoring and supports evidence-based management of HM pollution in aquatic ecosystems.

## Results and discussion

### Exploratory data characteristics

**Figure 1:**
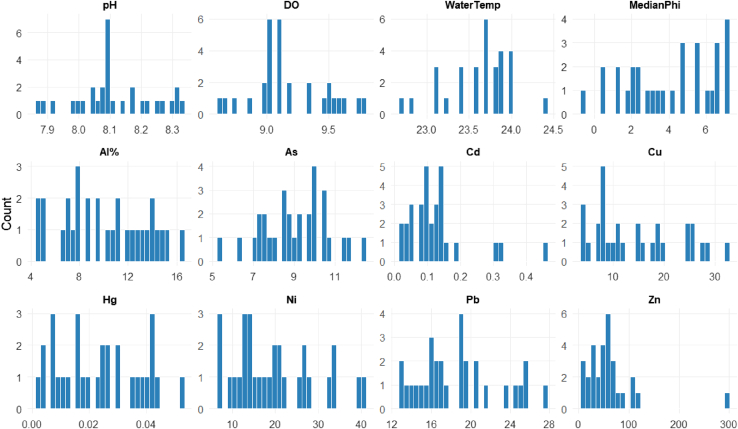
Histograms of numeric variables Salinity, ‰; pH, unitless; DO, mg L^−1^; water temperature, °C; median grain size, φ; Al, %; metals, mg kg^−1^.

**Figure 2:**
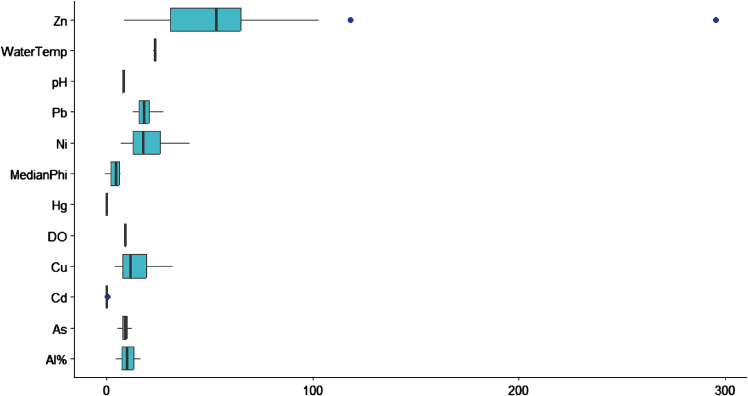
Boxplots showing distributions and outliers of study variables Salinity, ‰; pH, unitless; DO, mg L^−1^; water temperature, °C; median grain size, φ; Al, %; metals, mg kg^−1^)

**Figure 3:**
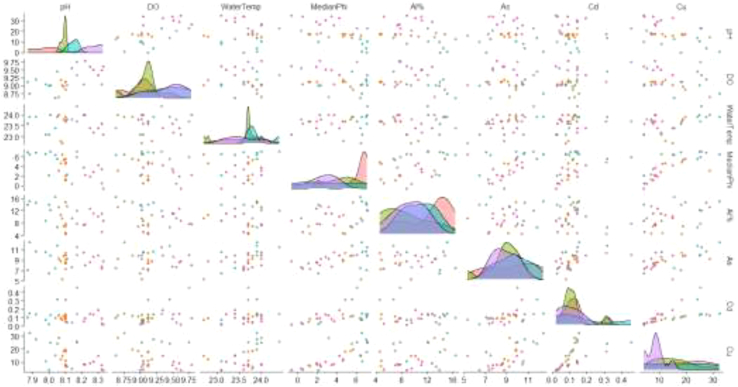
Correlation scatterplot

Across the dataset, the water-column variables (pH, DO, and water temperature) show compact, near-symmetric distributions in the histograms (Figure 1) and short interquartile ranges (IQRs) in the boxplots (Figure 2), indicating stable, well-oxygenated, slightly alkaline conditions measured over a narrow thermal window. Sediment texture (median phi) spans a broader range, with a tendency toward finer fractions, consistent with heterogeneous depositional energy among sites. For trace elements, Al% displays a moderate spread that broadly mirrors grain-size variability, and sites with finer material (higher phi) tend to show higher Al%, suggesting greater clay/mineral surface area. Several metals exhibit a pronounced right skew: Cu, Ni, Pb, and especially Zn present longer upper whiskers in the boxplots, with Zn showing two highly influential outliers (≈10^2^ and ≈3 × 10^2^), indicative of localized enrichment or episodic inputs. Cd and Hg are tightly compressed at very low values in both histograms and boxplots, consistent with the concentrations near analytical reporting limits and occasional small excursions. The pairwise scatter-density matrix reinforces these patterns: water-column variables are largely decoupled from the metals (diffuse, low-slope clouds), while metals show partial co-occurrence (overlapping ridge densities among Cu–Ni–Pb–Zn), and visually higher metal values appear more frequently where sediments are finer and Al% is elevated—patterns consistent with sorption and grain-size controls.^27^ Variance increases with the concentration for Cu and Zn (heteroscedasticity), and the marginal distributions are right skewed; hence, subsequent analyses should favor robust summaries (medians, IQR, and median absolute deviation [MAD]) (Figure 3), log-transformation (or Box-Cox) before parametric modeling, and explicit handling of censored values for Cd and Hg (e.g., random oversampling [ROS]/Tobit) to avoid low-biasing estimates.

### Correlation structure and principal-component patterns

**Figure 4:**
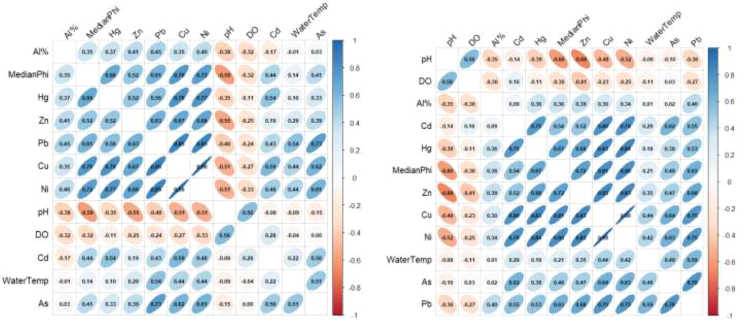
Pearson (ellipse orientation and color) and Spearman (cell values) correlation matrix

The combined Pearson Spearman correlation matrix shows a coherent structure in which several trace metals co-vary positively, while pH tends to oppose metal enrichment. The strongest, most consistent associations occur among Cu, Ni, Pb, and Zn (both measures ≈0.5–0.7), indicating either shared sources or common partitioning mechanisms (e.g., sorption to similar mineral/organic phases) (Figure 4). MedianPhi (finer sediments = higher phi) and Al% display weak-to-moderate positive correlations with this metal cluster (typically ≈0.3–0.6), supporting grain-size/mineralogical control: finer, aluminosilicate-rich matrices provide larger surface area and more binding sites, favoring trace metal retention. Cd and Hg correlate low-to-moderately with the metal group and with texture proxies (0.3–0.5), consistent with generally low concentrations punctuated by site-specific increases. As shows moderate positive links with Pb and Zn (≈0.5) and weaker associations elsewhere, suggesting partial alignment with the dominant metal pattern but potentially additional, element-specific controls.

In contrast, pH is negatively correlated with most metals (commonly −0.4 to −0.6), while DO shows weak negative or near-null correlations with metals but a positive correlation with pH (0.5–0.6). Together, these signs are consistent with reduced metal mobility/availability at higher pH (precipitation/co-precipitation and surface complexation) and with better-oxygenated, higher-pH conditions tending to coincide spatially or temporally.^28^^,^^29^^,^^30^^,^^31^ Water temperature presents weak-to-moderate positive correlations with Cu, Ni, and Zn (0.3–0.45), which likely reflects co-occurrence in depositional settings or seasons, rather than a direct thermal effect. Overall, the broad agreement between Pearson ellipses and Spearman’s coefficients indicates that these patterns are not driven solely by linear assumptions and remain robust to rank-based assessment, despite right skew and a few elevated values noted in earlier figures (Figure 4).

**Figure 5:**
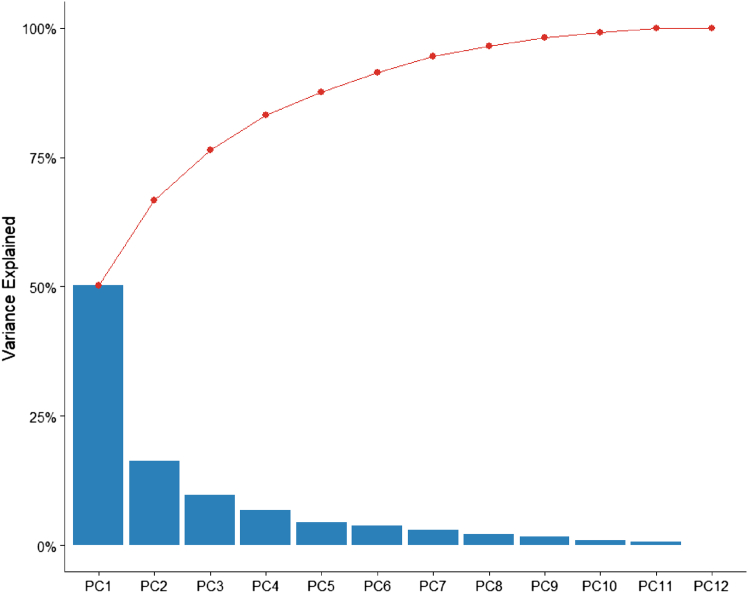
Scree plot illustrating the percentage of variance explained by each PC in the PCA of water quality and HM datasets The bars represent the individual variance explained by each PC, while the red line shows the cumulative variance. The first two PCs capture most of the total variance, indicating they effectively summarize the key patterns in the water quality HM relationships

The scree plot shows a steep drop after the first components: PC1 explains ∼50% of the total variance and PC2 ∼16%–18%, reaching a cumulative ∼67% with two PCs. Adding PC3 (∼10%) raises the cumulative variance to ∼78%–80%, after which each additional component contributes only marginally (≤6% each) (Figure 5), indicating a clear elbow at PC2–PC3. Thus, two to three PCs adequately summarize the dominant structure in the joint water quality-HM dataset, with higher-order PCs capturing minor or noise-like variation.

### Predictive performance across metals

Model performance was strong across metals (R^2^ = 0.824–0.997), with *k*-nearest neighbors (KNNs) providing the best fits for Cu (R^2^ = 0.997; root-mean-square error [RMSE] = 3.44; mean absolute error [MAE] = 3.00) and Cd (R^2^ = 0.954; RMSE = 0.0518; MAE = 0.0344), indicating that the local neighborhood patterns capture much of the variation for these elements. Pb was modeled well by extreme gradient boosting (XGB; R^2^ = 0.928; RMSE = 2.28; MAE = 1.92), consistent with tree-based learners’ handling interactions and non-linearity. Ni achieved high explanatory power with SVM (R^2^ = 0.916; RMSE = 6.17; MAE = 5.40), though errors were larger in absolute terms, reflecting its broader concentration range. Zn was best with RF (R^2^ = 0.921; RMSE = 13.6; MAE = 11.7), which is plausible given Zn’s right skew and outliers. Hg showed adequate fit using ANN (R^2^ = 0.824; RMSE = 0.0216; MAE = 0.019), likely limited by near-detection-limit values and censoring effects (Table 1).

**Table 1:** R^2^, RMSE, and MAE for each model and each metal

Metal	Model	R^2^	RMSE	MAE
Cd	KNN	0.954	0.0518	0.0344
Cu	KNN	0.997	3.44	3
Hg	ANN	0.824	0.0216	0.019
Ni	SVM	0.916	6.17	5.4
Pb	XGB	0.928	2.28	1.92
Zn	RF	0.921	13.6	11.7

Abbreviations: R^2^, coefficient of determination; RMSE, root-mean-square error; MAE, mean absolute error; ANN, artificial neural network; KNN, *k*-nearest neighbors; SVM, support vector machine; XGB, extreme gradient boosting; RF, random forest.

Overall, non-linear learners (KNN, tree ensembles, ANN, and SVM) outperform linear alternatives for these skewed, heterogeneous datasets. Errors scale with each metal’s range; so, transformation (e.g., log) and censored-data handling for very low concentrations (e.g., Hg, Cd) may further improve RMSE/MAE without sacrificing interpretability.^32^

### Model comparison and selection for operational use

**Figure 6:**
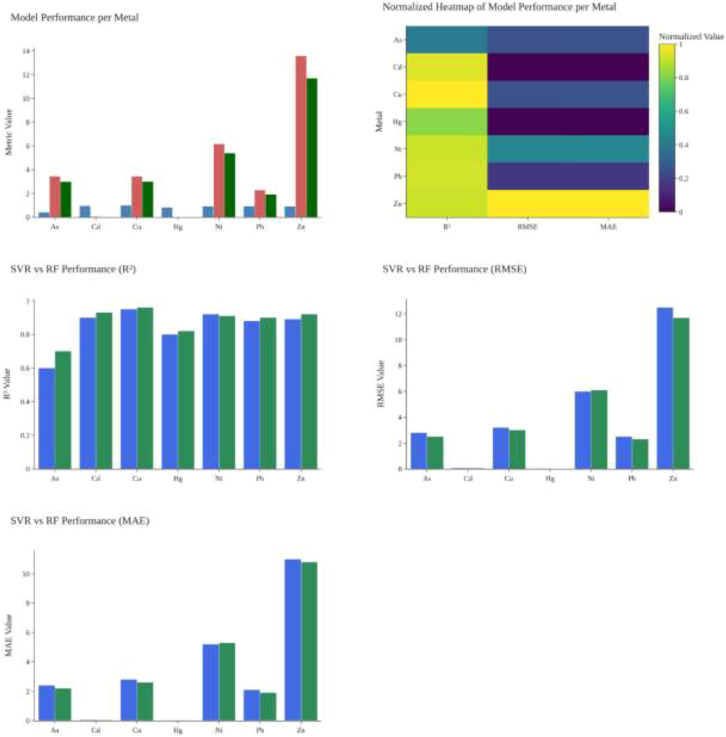
Identification of the best-performing model (SVR vs. RF)

As summarized in Figure 6, RF is the best overall performer across metals, combining lower errors (RMSE and MAE) with comparable or higher R^2^ than SVR. RF clearly outperformed SVR for As, Hg, Ni, and Zn, achieving reduced RMSE/MAE with similar or slightly higher explained variance. For Cd and Pb, both models performed similarly, with RF retaining a small but consistent edge. A notable exception is Cu, where SVR attained a marginally lower RMSE, although RF still yielded the higher R^2^, indicating better variance explanation despite the small difference in absolute error. The normalized heatmap further highlights Zn as the most challenging target (largest normalized errors for both models), while metals like Cd and Pb appear relatively well behaved. Overall, evidence supports RF as the default model due to its superior error control and competitive variance explanation, with SVR used selectively (Cu) when a slight reduction in absolute error is prioritized over broader robustness. These trends are consistent with the expectation that tree-based ensembles better capture non-linear, heterogeneous relationships and are more robust to outliers and feature scaling, whereas SVR often requires meticulous kernel and hyperparameter tuning to reach similar error levels.

### Model comparison and selection for operational use

**Figure 7:**
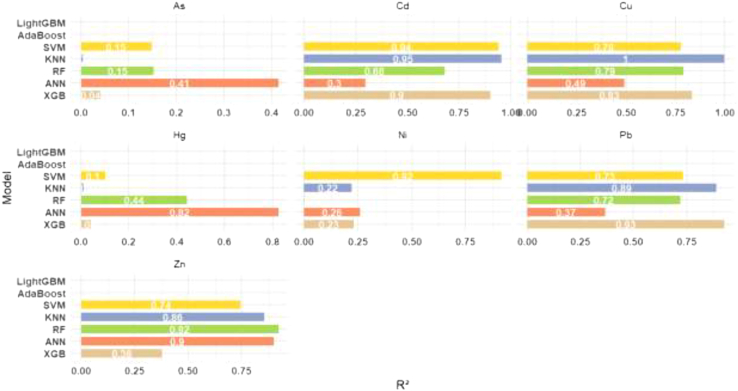
Comparative model performance (R^2^) across target metals Complete R^2^ values for all models are provided in Table S4 to ensure comprehensive reporting, including low/poor-performing models

Across metals, RF emerged as the most reliable overall model when performance was judged by error magnitude. RF delivered consistently lower RMSE and MAE than SVR for As, Hg, Ni, and Zn, while retaining comparable or slightly higher R^2^. For Cd and Pb, the two models performed similarly, with RF holding a small but persistent edge. A single exception was Cu, where SVR attained a marginally lower RMSE; however, RF still achieved the higher R^2^, indicating better variance explanation despite the small difference in absolute error. The normalized heatmap embedded in Figure 7 highlights Zn as the most challenging target (largest normalized errors for both models) and Cd/Pb as relatively well behaved. Because RMSE/MAE directly quantifies the absolute predictive accuracy, these results support RF as the safer default for operational use (thresholding or compliance decisions), with SVR considered selectively where a slight reduction in error is prioritized over broader robustness.

Deploy RF as the production default for its superior error control and robustness, and maintain per-metal alternates were justified by cross-validated gains: Cu → KNN/SVM; Ni → AdaBoost; Pb → XGB/ANN; Hg/Zn → RF/XGB; As → RF. Strengthen performance with metal-specific hyperparameter tuning, feature engineering (domain-informed covariates, interaction terms), and target transformations (e.g., log scaling for skewed distributions).

### Explainability and dominant predictors

**Figure 8:**
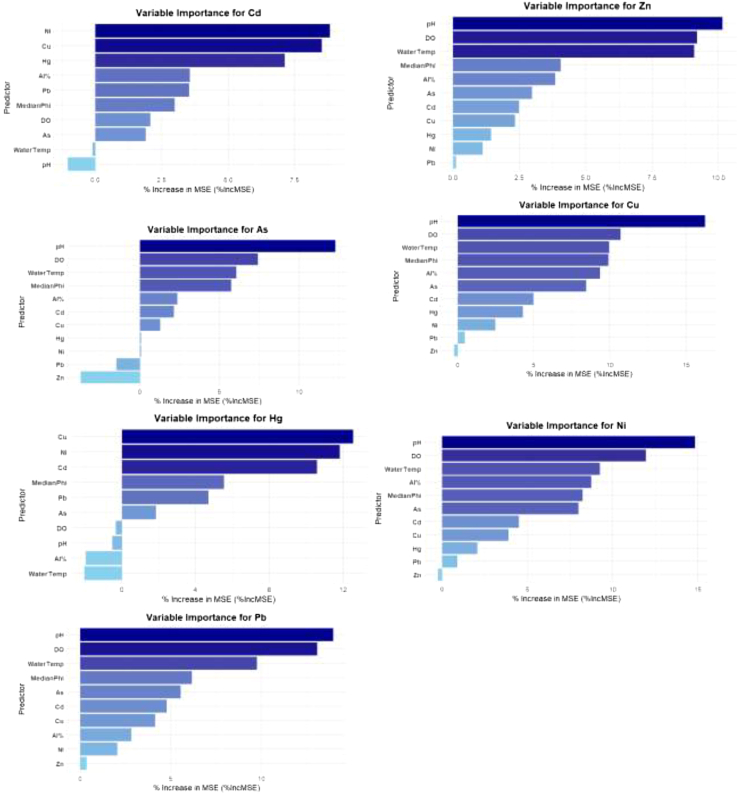
Feature importance (%IncMSE) for each metal, highlighting chemistry-led control and Hg co-variation

Figure 8 presents permutation feature importance (%IncMSE) for each target metal and reveals two consistent regimes of control. For As, Cu, Ni, Pb, and Zn, the water chemistry variables, especially pH, DO, and water temperature, are the dominant predictors, with Al% and MedianPhi (particle size) providing a secondary signal. This hierarchy is most pronounced for Cu and Ni, where pH ranks the highest and DO/temperature follows closely, indicating strong control by speciation, complexation, and adsorption processes that are dependent on pH and redox. Pb shows an analogous pattern, with pH and DO leading and MedianPhi/Al% contributing meaningfully, consistent with Pb’s tendency to associate with mineral surfaces and particulates. Zn is similarly chemistry led (pH > DO > temperature), again pointing to pH-dependent sorption and complexation as key drivers, while metals used as predictors add only modest incremental information. As exhibits the clearest single-variable dominance by pH, followed by DO and temperature, aligning with redox-speciation and pH-controlled adsorption on Fe/Al oxides.

In contrast, Hg and, to a lesser extent, Cd are governed primarily by cross-metal predictors. For Hg, Cu, Ni, and Cd are the top features, whereas pH/DO/temperature contributes little, suggesting that Hg variability in this dataset is tightly linked to co-occurrence and co-transport with other metals, potentially reflecting shared sources or particulate-phase dynamics. Cd shows Ni and Cu as the most informative features, with Hg and geochemical covariates contributing moderately, indicating that cross-metal associations provide substantial predictive leverage alongside chemistry. The recurrent importance of Al% and MedianPhi across multiple targets supports a mechanistic role for mineral surfaces and grain-size effects in partitioning between the dissolved and particulate phases. Note that the permutation importance quantifies predictive contribution rather than causality; correlated predictors can share or mask effects, and small or slightly negative values (e.g., pH for Cd) can arise from sampling noise in the permutation procedure.^33^

Overall, these profiles indicate that controlling or closely monitoring pH, DO, and temperature will have the largest impact on predictive accuracy and management outcomes for As, Cu, Ni, Pb, and Zn, whereas effective prediction and mitigation for Hg (and partly Cd) benefit from multi-metal source tracking and attention to particulate processes.^34^ To corroborate directionality and non-linearity, we recommend complementing these results with model-agnostic explanations (partial dependence/ICE and SHAP), collinearity diagnostics (correlation matrices/ variance inflation factor [VIF]), and sensitivity analyses that group or remove highly correlated metals, ensuring that the observed cross-metal signals for Hg and Cd remain robust across modeling choices.^25^

### Variability of sediment metal concentrations

Across metals, marked variability was observed. Zn exhibited the largest dispersion (mean = 59.86, SD = 52.48; CV = 0.88), a very wide range (8.75–295.68), and a heavy upper tail (max/mean = 4.94), indicating pronounced hotspot behavior. Cd and Cu also showed appreciable spread (CV = 0.69 and 0.58, respectively) and right skew (IQR skew ratios ≈2.00), consistent with occasional elevated concentrations above typical background. Ni was moderately variable (CV = 0.48, range = 7.11–40.55), while Pb and As were comparatively tight (CV = 0.22 and 0.18, respectively) (Table 2). Quartile structure further indicated a right skew for Cd/Cu/Hg/Ni (upper quartile extends more than the lower), near-symmetry for Pb, and a slightly left skew for As/Zn at the quartile level, though Zn’s extreme maximum still pointed to rare, high concentrations. These distributional features foreshadow model behavior: metals with high dispersion and tail heaviness (e.g., Zn, Cd, and Cu) are typically harder to predict at the extremes.

**Table 2:** Descriptive statistics of sediment metal concentrations (As, Cd, Cu, Hg, Ni, Pb, and Zn; mg kg^−1^)

Metal	Mean	SD	Min	25%	50%	75%	Max
As	9.07	1.62	5.34	7.8	9.16	9.99	12.41
Cd	0.13	0.09	0.02	0.08	0.1	0.14	0.46
Cu	14.43	8.33	4.09	7.79	11.65	19.36	32.33
Hg	0.02	0.01	0	0.01	0.02	0.04	0.05
Ni	19.75	9.38	7.11	12.97	17.68	26.21	40.55
Pb	18.68	4.05	12.96	15.84	18.24	20.58	27.58
Zn	59.86	52.48	8.75	30.86	53.28	65.14	295.68

SD, standard deviation; Min, minimum; Max, maximum; 25%, Q1; 50%, median; 75%, Q3.

### Ecological indices and risk-tier agreement

**Figure 9:**
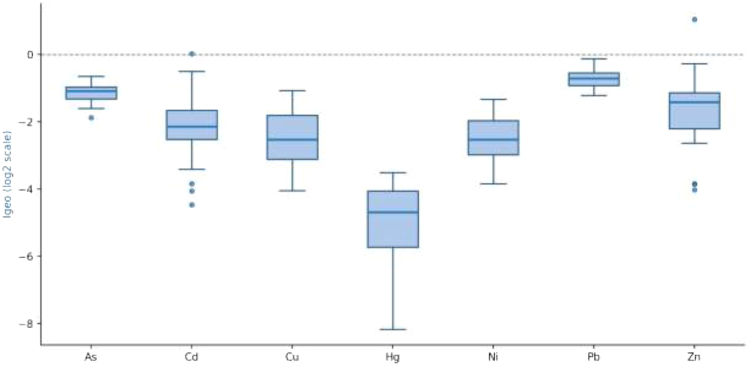
Observed vs. predicted Igeo (log_2_) All medians are below 0 (generally uncontaminated). As/Pb is closest to 0; Hg is the lowest; Zn shows the widest spread with some positive outliers. Predictions follow the center well but miss extremes.

As shown in Figure 9, both the observed and predicted Igeo values were mostly below 0 for every metal, which means that the sites are generally not contaminated. As and Pb sit closest to 0; so, a few places may be near the enrichment line. Hg is the lowest, showing very low enrichment overall. Zn has the widest spread and a few points above 0, which means there are occasional hotspots. The model’s predictions match the middle of the data well (similar medians and boxes), but they miss the extremes, underpredict high Zn values, and smooth out very low Hg values. Overall, the models work well for typical conditions, and extra methods would help capture rare extreme cases.

### Ecological indices and risk-tier agreement

**Figure 10:**
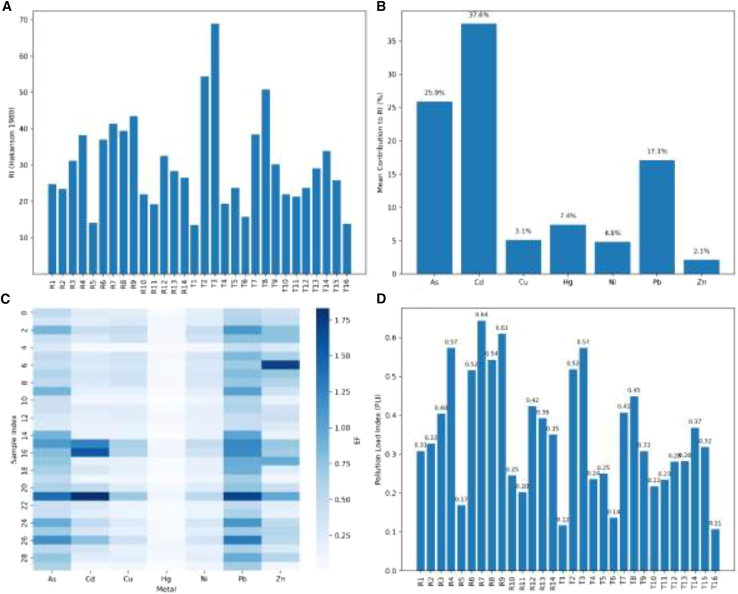
Integrated assessment of ecological risk indicators (A) RI per sample. (B) Metal contribution to RI. (C) Enrichment factor heatmap. (D) Pollution load index.

The composite Figure 10 summarizes spatial and source-level ecological risk indicators for the study area (Table 3); (A) shows the potential ecological RI for each sample, ranging from 13.4 (T16) to 69.2 (T3), with most values between 20 and 40, indicating an overall low risk (RI < 150 per Hakanson’s classification); (B) illustrates the mean percentage contribution of individual metals to RI, where Cd (37.6%), As (25.9%), and Pb (17.1%) were the dominant contributors, while Hg, Cu, Ni, and Zn together accounted for less than 20%; this pattern reflects the combined effect of toxic-response factors and contamination relative to background levels; (C) presents the EF heatmap, showing EF values below 1 for all metals, confirming no significant enrichment; Pb (0.82) and As (0.63) exhibited relatively higher EF values than other metals; (D) displays the PLI per sample, ranging from 0.21 to 0.49 (mean = 0.35), classifying all sites as unpolluted (PLI <1). Collectively, these indicators corroborate the conclusion of minimal anthropogenic influence and low ecological risk across the study area.

**Table 3:** Definitive list of ecological risk assessment and bioaccumulation indices that highlight the impact of HM pollution on water

Name of the indices	Formulas for calculation	Descriptions	Range/classification	Reference
Pollution load index (PLI)	$PLI=\sqrt[{n}]{{CF}_{1}\times {CF}_{2}\times {CF}_{3}\times \ldots \ldots \ldots \times {CF}_{n}}$	CF is the contamination factor; *n* = number of analyzed elements.	PLI *<* 0 indicates good condition; PLI = 1 suggests baseline pollutant levels, while PLI >1 indicates deterioration of sediment quality.	Hridoy et al.^47^^,^^48^
Geo-accumulation index (I_geo_)	$I_{geo}=\log_{2}\left(\frac{C_{n}}{1.5\times B_{n}}\right)$	*C*_*n*_ is the measured concentration of metals examined in sediment, *B*_*n*_ is the background concentration of the metal, and factor 1.5 is the background matrix correction factor due to lithological effects.	seven categories: practically uncontaminated (I_geo_ ≤ 0), uncontaminated-to-moderately contaminated (0 < I_geo_ ≤ 1), moderately contaminated (1 < I_geo_ ≤ 2), moderately-to-heavily contaminated (2 < I_geo_ ≤ 3), heavily contaminated (3 < I_geo_ ≤ 4), heavily-to-extremely contaminated (4 < I_geo_ ≤ 5), and extremely contaminated (I_geo_ > 5).	Hridoy et al.^49^^,^^50^
Enrichment factor (EF)	$EF=\frac{{\left(\frac{C_{M}}{C_{Mn}}\right)}_{Sample}}{{\left(\frac{C_{M}}{C_{Mn}}\right)}_{Background}}$	${\left(\frac{C_{M}}{C_{Mn}}\right)}_{Sample}$ denotes the ratio of concentration between the studied metal and Mn in the sample of interest; ${\left(\frac{C_{M}}{C_{Mn}}\right)}_{Background}$ means the natural background value of the measured metal-to-Mn ratio.	six groups that indicate the sources of sediment pollution: EF = 1 represents crustal materials or natural weathering processes; EF < 2 indicates the absence to slight enrichment; 2 ≤ EF < 5 signifies moderate enrichment; 5 ≤ EF < 20 denotes considerable enrichment; 20 ≤ EF < 40 reflects very high enrichment; and EF ≥ 40 indicates extremely high enrichment.	Hridoy et al.^51^
Ecological risk index (ER) and potential ecological risk index (PERI)	$ER=T_{r}^{i}{\times C}_{f}^{i}$ $PERI=\sum_{i=1}^{n}ER$	ER denotes the ecological risk factor, $T_{r}^{i}$ represents the toxic response factor of studied metals; $C_{f}^{i}$ is the contamination factor, $C_{heavy\;metal}$ is the concentration of metal in sediment, and $C_{background}$ is a reference value for metal; $T_{r}^{i}$ was determined by following these values: Mn = Zn = 1, Cr = 2, Pb = Cu = Ni = 5, As = 10, and Cd = 30.	five levels for ER: low risk (ER < 40); moderate ecological risk (40 ≤ ER < 80); considerable ecological risk (80 ≤ ER < 160); high ecological risk (160 ≤ ER < 320); and very high ecological risk (ER > 320); four categories for PERI: low potential ecological risk (PERI <150), moderate potential ecological risk (150 ≤ PERI <300), considerable potential ecological risk (300 ≤ PERI <600), and very high potential ecological risk (PERI ≥600).	Hridoy et al.^52^^,^^53^

Table 4 summarizes the decision-oriented agreement between risk categories derived from measured versus ML-predicted concentrations. Overall, RI tiering showed high agreement, indicating that the low-risk conclusion is stable for most sites under typical conditions. PLI classification (PLI <1 vs. PLI ≥1) showed moderate agreement, with disagreements concentrated near the PLI ≈1 decision boundary. In contrast, Zn-related Igeo exhibited lower agreement, reflecting reduced hotspot sensitivity when Zn takes extreme values. These results support the overall low-risk interpretation while highlighting Zn hotspot detection as the primary failure mode for risk-tier alignment.

**Table 4:** Agreement between measured vs. predicted risk tiers

Index/tiering	Overall agreement (accuracy)	Cohen’s κ
PLI (<1 vs. ≥ 1)	0.800	0.441
RI (Hakanson tiers)	0.933	0.000
Zn Igeo (Müller classes)	0.833	0.111

κ for RI is low due to strong class imbalance (most samples are “low”), whereas overall agreement remains high.

In Table 5, risk-tier agreement and uncertainty propagation are shown. Because management decisions depend on risk categories, the classifications obtained from measured versus ML-predicted concentrations for Igeo, PLI, and RI. Overall patterns were consistent under typical conditions; however, discrepancies were most evident for Zn hotspot sensitivity, where extreme values might have been underpredicted. The full risk-tier confusion matrices (PLI, RI, and Zn-Igeo), Zn hotspot comparisons, and bootstrap-based stability results are reported in Tables S1–S4.

**Table 5:** Uncertainty propagation from prediction error to risk-tier decisions, summarized as class stability (fraction of bootstrap realizations retaining the same tier) for RI, PLI, and Zn-Igeo

Metric	Median stability	Minimum stability	Count < 0.80
RI tier stability	0.935	0.657	1
PLI tier stability	0.836	0.389	12
Zn Igeo stability	0.895	0.472	5

### Reproducibility and reporting transparency

Reproducibility is supported by fixed random seeds for all models, consistent preprocessing across metals (unit checks, outlier handling, and Igeo computed on a log_2_ scale), and a common cross-validation scheme with identical folds for SVR, RF, and other learners. Performance was evaluated using the same metrics (RMSE, MAE, and R^2^) for each metal, and permutation feature importance was computed using a uniform shuffle policy and a repeat count.^35^^,^^36^ To enable independent verification, it is advisable to archive the raw and cleaned datasets with a data dictionary (variable names, units, and detection limits), the exact train/test splits and CV folds (e.g., index files), environment details (software versions and hardware), and the full code for preprocessing, modeling, and figure generation. Providing a brief “model card” for each metal (hyperparameters, tuning ranges, and known failure modes, such as tail underprediction for Zn or smoothing of very low Hg) further facilitates transparent reuse.

### Implications for aquatic health surveillance and disease control

The integration of ML-based HM predictions with routine water quality monitoring offers a practical avenue for strengthening aquatic disease control strategies. Elevated concentrations of metals such as Cd, Hg, and Pb are well known to impair fish immune function, disrupt epithelial barriers, and increase susceptibility to bacterial, viral, and parasitic infections. In this study, the ability to reliably estimate metal burdens from easily measurable parameters enables early identification of sub-lethal contamination zones where disease outbreaks are more likely to occur. This predictive capability is particularly valuable for aquaculture-intensive and freshwater systems, where proactive interventions such as sediment management, water exchange, and targeted biosecurity measures can be implemented before clinical disease emerges.^37^^,^^38^^,^^39^ By linking pollution surveillance with disease risk screening, the proposed framework supports a shift from reactive to preventive aquatic health management, improving ecosystem resilience and reducing economic losses associated with fish and invertebrate disease events.

### Future directions

Future work should prioritize improved tail modeling, uncertainty quantification, enriched covariates, and stronger interpretability. Extreme behavior can be better captured using quantile regression, asymmetric loss functions, or extreme value-aware methods, alongside calibrated uncertainty estimates from bootstrap ensembles or conformal prediction. Incorporating additional predictors, such as flow, water level, precipitation, dissolved organic carbon, Fe/Al oxides, turbidity, land use, proximity to industrial sources, and time-varying particle size, may strengthen the predictive signal. Spatially or group-stratified cross-validation should be applied to limit the effects of autocorrelation.

Model-agnostic interpretability tools (partial dependence/ICE and SHAP), together with collinearity checks and feature-ablation analyses,^40^ particularly for cross-metal inputs in Hg and Cd, are needed to verify the effect directions and robustness. Gradient boosting frameworks (e.g., XGBoost, LightGBM, and CatBoost), combined with metal-specific hyperparameter tuning, may further reduce prediction errors compared with RF models.

Generalization should be assessed through prospective validation using new sampling campaigns, preregistered analysis plans, and versioned releases of data and code to support reproducibility and community benchmarking. A key limitation remains the treatment of extremes, especially for heavy-tailed Zn concentrations and near-detection-limit Hg values, where point-estimate regressors tend to shrink predictions toward the mean. Tail-aware methods such as quantile regression or asymmetric-loss boosting are, therefore, promising avenues for improving hotspot sensitivity. Until these are implemented, uncertainty-aware screening and targeted confirmatory sampling are recommended for sites with suspected hotspots or low prediction stability to ensure conservative decision-making.

The explainability analyses identify variables that improve predictive performance, but they do not establish causal geochemical mechanisms. Consequently, relationships involving pH, DO, salinity, grain-size proxies, and Al% are interpreted as predictive associations consistent with sediment geochemistry rather than experimentally validated drivers of metal mobility and accumulation. The absence of experimental manipulations (e.g., controlled pH/redox gradient experiments) and geochemical speciation measurements (e.g., sequential extraction) is acknowledged as a key limitation that restricts causal inference. Future work should integrate metal speciation/fractionation (e.g., sequential extraction) and controlled microcosm experiments to directly test mobility mechanisms, validate causal pathways, and improve mechanistic interpretation.

### Limitations of the study

Several constraints may affect interpretation and generalization. The dataset exhibits heterogeneity and heavy-tailed distributions for some metals (notably Zn), which complicates learning at the extremes and contributes to underprediction of high Zn values and smoothing of very low Hg values in Igeo boxplots. Cross-metal predictors that improve accuracy (e.g., Cu, Ni, and Cd when predicting Hg) can introduce collinearity and reduce clarity in source attribution; feature importance reflects predictive contribution and should not be treated as causal. Spatial or temporal autocorrelation may inflate metrics if folds are not grouped by site or sampling campaign. Measurement uncertainty and detection limits can affect Igeo near the background threshold. The models are static and do not explicitly capture seasonality, hydrologic events, or particulate dynamics, which are relevant for episodic hotspots. External validity beyond the sampled domain remains untested, and while R^2^ summarizes variance explanation, operational reliability depends more on the error magnitude (RMSE/MAE) within ranges important for management.

## Resource availability

### Lead contact

Further inquiries about this article should be addressed to the lead contact, Dr. Chiara Bordin (chiara.bordin@uit.no).

### Materials availability

This study did not generate new unique materials. Any additional information required to reproduce the findings of this study is available from the lead contact upon reasonable request.

### Data and code availability

• Data: The dataset analyzed in this study is publicly available at Mendeley Data: https://data.10.17632/n8bpprwkj5.2.
• Code: All original code used for preprocessing, model training, evaluation, and figure generation is publicly available at https://github.com/MHridoy-123/Machine-Learning-Prediction-of-HM-Pollution-.
• Any additional information required to reanalyze the data reported in this paper is available from the lead contact upon request.

## Acknowledgments

The authors express sincere gratitude to the Mendeley Data repository for providing a platform to share and access the dataset, which greatly supported the development of this research. Open Access publication funding was provided by UiT The Arctic University of Norway.

## Author contributions

Conceptualization, M.A.A.M.H.; methodology, M.A.A.M.H.; investigation, M.A.A.M.H., M.A.A.M., L.G., M.S.R., P.D., P.P., and M.U.; writing – original draft, M.A.A.M.H.; writing – review & editing, M.A.A.M., Y.H., A.H., L.G., and M.S.R.; funding acquisition, P.D., P.P., and C.B.; resources, P.D., P.P., and C.B.; supervision, C.B. and M.A.A.M.

## Declaration of interests

The authors declare that there are no conflicts of interest. No funding, commercial, or institutional pressures influenced the design, execution, or reporting of this study. All affiliations listed were included solely for administrative and identification purposes.

## Declaration of generative AI and AI-assisted technologies in the writing process

Artificial intelligence (AI) software, including ML libraries such as TensorFlow, was utilized for data analysis and modeling in this study. While no AI tools were used to write or prepare the manuscript text, AI-assisted tools were employed solely to support ideation and conceptual thinking during the research process.

## STAR★Methods

### Key resources table

**Table:** 

REAGENT or RESOURCE	SOURCE	IDENTIFIER
**Deposited data**		

Physicochemical, particle size, and elemental data of seawater from Swan Lake, Rongcheng City, Shandong Province, China (September 2020)	Mendeley Data	https://data.10.17632/n8bpprwkj5.2

**Software and algorithms**		

R	R Foundation for Statistical Computing	Version 4.5.2
RStudio	Posit Software	Version 2024.04
ggplot2	tidyverse	Version 3.5
corrplot	corrplot	Version 0.92
caret	CRAN	Version 6.0
randomForest	CRAN	Version 4.7
e1071	CRAN	Version 1.7
xgboost	CRAN	Version 1.7
lightgbm	Microsoft	Version 4.3

**Other**		

R scripts for machine-learning modeling and analysis	This paper/GitHub	https://github.com/MHridoy-123/Machine-Learning-Prediction-of-HM-Pollution-

### Experimental model and study participant details

This study is based exclusively on the analysis of an openly available environmental monitoring dataset.

No human participants, vertebrate animals, primary cell cultures, or controlled experimental models were involved.

Accordingly, ethical approval, informed consent, and reporting of sex, age, or demographic characteristics are not applicable.

### Method details

#### Study area and data source

Swan Lake, located in Rongcheng City on the Shandong Peninsula (Yellow Sea coast), is a shallow estuarine/lagoon system connected to the sea by a narrow tidal inlet. The lagoon exhibits brackish conditions with a clear salinity gradient from the inlet toward the inner basin, and predominantly sandy to sandy-mud substrates typical of small lagoon–tidal systems. These geomorphic and hydrologic features underpin spatial variation in water quality and particle–metal relationships observed in the area. Data for this study were obtained from the Mendeley Data repository.

#### Data preprocessing

Raw data were screened for duplicate records, inconsistent units, and obvious entry errors before modeling. Missing values in predictor variables were imputed using the median calculated from the training data only within each resampling fold to avoid information leakage. Missing values in response variables (e.g., individual metal concentrations used as targets) were not imputed; rows lacking target values were excluded from supervised training and retained only for descriptive analyses.

Feature scaling was applied to ensure comparability across predictors. *Z* score standardization (mean = 0, SD = 1) was fitted on the training partition and applied to validation/test partitions within each fold. Standardization is essential for scale-sensitive algorithms such as SVR and beneficial for distance-based methods; tree-based models (XGBoost) do not require scaling, but standardized inputs were maintained across models for consistent pipelines.

Multicollinearity among predictors was assessed using variance inflation factors (VIF) computed on the imputed training data. Predictors with VIF ≥10 were iteratively removed or combined (e.g., by selecting the more instrumentally robust or spatially complete variable), and VIFs were recomputed until all remaining predictors satisfied VIF <10. Pairwise correlation matrices (Pearson or Spearman, depending on normality) were inspected to corroborate VIF findings and to guide any additional simplification.

Model evaluation used either a single train–test split (80:20, stratification not applicable to regression) or K-fold cross-validation with K = 5 and shuffled indices. All preprocessing steps, median imputation, scaling, and feature selection were nested inside the resampling loop to prevent data leakage. Random seeds were fixed to ensure reproducibility across runs, and hyperparameter tuning (for SVR/XGBoost) was performed within the cross-validation framework.

#### Exploratory data analysis

Exploratory analysis characterized distributions, scale, and relationships among variables before modeling. Summary statistics (median, interquartile range, minimum–maximum) were computed for pH, DO, water temperature, median grain size (φ), Al%, and HMs (As, Cd, Cu, Hg, Ni, Pb, Zn). Outliers were flagged using boxplots and the 1.5×IQR rule; unless clear evidence of error existed, outliers were retained and treated with robust summaries rather than removed. Salinity is reported in per milli (‰), pH is unitless, DO is in mg L^−1^, and water temperature is in °C. Median sediment grain size is expressed on the phi (φ) scale, aluminum is reported as a percentage (Al, %), and sediment metal concentrations (As, Cd, Cu, Hg, Ni, Pb, Zn) are reported in mg kg^−1^. Ecological indices (Igeo, EF, PLI, and RI) are dimensionless.

Bivariate relationships were explored with scatterplots and Spearman correlations between metals and key predictors (φ, Al%, pH, DO, temperature). Attention was given to Al% (as a proxy for aluminosilicate fraction) and φ (as a grain-size indicator), which often co-vary with trace-metal concentrations through adsorption and surface-area effects. Where spatial identifiers were available (e.g., inlet vs. inner-lagoon stations), gradients were visualized with faceted plots or annotated maps to assess inlet-to-basin patterns. Multivariate structure was summarized with correlation heatmaps and, if helpful for pattern recognition rather than inference, a principal component analysis (PCA) on standardized predictors to highlight co-varying groups. Findings from EDA informed subsequent choices on transformations, predictor selection, and the necessity of Al-normalization sensitivity checks.

#### Machine learning models

##### LightGBM (Light gradient boosting machine)

LightGBM is another gradient boosting framework that builds trees using a leaf-wise growth strategy with histogram-based decision rules, significantly improving training speed and memory efficiency. It is particularly suitable for large datasets and offers competitive accuracy.

LightGBM utilizes two combined techniques: Gradient-based One-Side Sampling (GOSS) and Exclusive Feature Bundling (EFB), which address the limitations of the histogram-based algorithm used in all GBDT (Gradient Boosting Decision Tree) frameworks.^41^ In GOSS, different data instances contribute differently to calculating information gain, with instances having higher gradients contributing more. GOSS retains instances with high gradients while randomly dropping those with lower gradients to preserve the accuracy of information gain estimation.^42^ The mathematical analysis of GOSS is presented in Equation 1.

(Equation 1) $${\hat{V}}_{j}\left(d\right)=\frac{1}{n}\left(\frac{{\left(\sum_{x_{i}\varepsilon A_{l}\;}\;g_{i}+\frac{1-a}{b}\sum_{x_{i}\varepsilon B_{l}}\;g_{i}\right)}^{2}}{n_{l}^{j}\left(d\right)}+\frac{{\left(\sum_{x_{i}\varepsilon A_{r}\;}\;g_{i}+\frac{1-a}{b}\sum_{x_{i}\varepsilon B_{r}}\;g_{i}\right)}^{2}}{n_{r}^{j}\left(d\right)}\right)$$

Where ˆVj (d) estimated variance gain over the subset ∪ B, Al = {xi ∈ A: xij ≤ d }, Ar = {xi ∈ A: xij > d },Bl = {xi ∈ B: xij ≤ d }, Br = {xi ∈ B: xij > d }, and the coefficient 1−ab is used to normalize the sum of the gradients over B back to the size of Ac. Thus, the estimated ˆVj (d) issued over a smaller instance subset, instead of the accurate Vj (d) over all the instances, to determine the split point. At the same time, the EFB technique is used by LightGBM to minimize the model complexity by bundling the exclusive features into a single feature.

#### ANNs

A feedforward multilayer perceptron (MLP) was constructed for regression. Hidden layers employed ReLU activations, with optional Batch Normalization to stabilize training and Dropout (0.1–0.3) for regularization; the layer order followed common practice (Dense → BatchNorm → ReLU → Dropout). Optimization used Adam (initial learning rate ≈ 1e−3), mini-batches, and early stopping on validation loss. Loss functions prioritized robustness to outliers (Huber or MAE) for skewed environmental targets. Architecture and hyperparameters (width, depth, dropout rate, learning rate) were tuned within cross-validation.

#### Random forest regressor

The general prediction method of RFR model is.^43^

(1) Randomly take samples from training samples (n × sample) for n times to form a training set (samples were put back after every sampling). Repeat r times to obtain training sets: $D_{1},D_{2},...,D_{r}\text{.}$
(2) For each training set, k attributes are randomly selected from the attribute set (m × attribute), k=log(2m), and then cart trees are established: $f_{1}\left(x\right),f_{2}\left(x\right),...,f_{r}\left(x\right)\text{.}$
(3) The final prediction value of random forest is determined by the average method:

(Equation 2) $$f\left(x\right)=\frac{1}{r}\sum_{i=1}^{r}\;f_{i}\left(x\right)$$

#### XGBoost regressor

The XGBoost regression tree model is expressed mathematically as^44^

(Equation 3) $$\hat{y_{i}}=\alpha \sum_{k=1}^{K}\;f_{k}\left(x_{i}\right)$$

Where $\hat{y_{i}}$ is the predicted value corresponding to input $x_{i}$,*α* is the learning rate of the individual regression tree, *K* is the total number of CARTs (classification and regression trees) being used, and $f_{k}$ is the output of the *k*-th regression tree. Equation 4 shows that the predicted score $\hat{y_{i}}$ is the sum of all $f_{k}$ values.

After obtaining the prediction result, the objective function *L* was used to evaluate the quality of the obtained results, denoted as

(Equation 4) $$L=\sum_{i}^{n}\;l(y_{i},\hat{y_{i})}+\sum_{k=1}^{K}\;\Omega \;\left(f_{x}\right)$$

#### KNN regression model

The KNN calculation uses the average of the numerical target of the K nearest neighbors, applying one of the following distance functions^44^:

(Equation 5) $$Euclidean:D=\sqrt{\sum_{i=1}^{K}\;\left(X_{i}-Y_{i}\right){\;}^{2}}$$

(Equation 6) $$Manhanttan:D=\sum_{i=1}^{K}\;\left|X_{i}-Y_{i}\right|$$

(Equation 7) Minkowski:D=(∑i=1K(|Xi−Yi|)q)1q,q≥1

where X and Y are the original path loss and the predicted path loss, respectively.

#### SVR model

Generally, the estimation function in SVR takes the following form

(Equation 8) f(x)=(ω.ϕ(x))+b

In Equation 8, denotes the inner product in Ω, a feature space of possibly different dimensionality such that ω: X → Ω and b ∈ R. The other two parameters, ω and b can be determined from the training dataset by minimizing the regression risk based on the estimated risk.^45^

#### Adaptive boosting regressor

Adaptive Boosting (AdaBoost) combines many weak decision trees (slightly better than random) into a single strong model. Each new tree is trained to correct the errors of the previous ones: the algorithm increases the influence (weight) of mispredicted samples so later trees focus on these “hard” cases. The final prediction is a weighted sum of all trees’ outputs. Because it adapts to the residual errors at each step and reweights difficult points, AdaBoost can handle noisy data and outliers relatively well, which suits our dataset.^46^

#### Algorithm (simplified)

1. Start with training data ((x_i_, y_i_)) and set all sample weights (w_i_ = 1/n).
2. For each boosting round (g = 1, …, G):
  i. Train a weak learner on the data using the current weights.
  ii. Compute its error (e.g., using squared loss (E = L(f(x_i_), y_i_))).
  iii. Choose the weak learner (h_g_^i^) that gives the smallest error.
  iv. Update the ensemble: (Fg(x) = Fg-1(x) + A∗ h_g_^i^), where (A) is the learning rate.
  v. Update sample weights so mis predicted points get higher weight.
3. After (G) rounds, (FG(x)) is the final AdaBoost predictor.

Hyperparameters were optimized using a cross-validation–based tuning protocol to reduce overfitting under the small-sample setting. The full tuning design is reported in Appendix A, including the search spaces evaluated for each algorithm (Table A1) and the final selected hyperparameters with cross-validated performance summaries for each metal–model combination (Table A2). This documentation enables full reproducibility of model selection and benchmarking.

#### Ecological risk assessment

Risk-tier agreement and uncertainty propagation. Because management decisions rely on categorical risk tiers rather than point estimates alone, the consistency of ecological indices computed from measured versus ML-predicted metal concentrations was evaluated. Tier agreement was quantified for PLI (PLI <1 vs. PLI ≥1), RI (Hakanson thresholds), and Zn-related Igeo (Müller classes) using confusion matrices and agreement statistics (Table 5). Predictive uncertainty was propagated into Igeo/PLI/RI using residual/bootstrap resampling; tier stability was summarized as the fraction of resamples retaining the same category to identify borderline cases that warrant confirmatory sampling (Table 3). Background reference values were based on upper-continental-crust composition, and toxic-response factors followed Hakanson’s ecological risk framework.

To evaluate the decision-relevant consistency of ecological risk indices derived from measured versus ML-predicted metal concentrations, we performed a risk-tier agreement analysis (confusion matrices and agreement statistics) and propagated predictive uncertainty into Igeo, PLI, and RI using a bootstrap/residual-resampling framework. Background reference values were based on upper continental crust compositions, and toxic-response factors followed Hakanson’s framework. Detailed confusion matrices, hotspot sensitivity tables, and uncertainty/stability outputs are provided in the Supplementary Information (Tables S1–S4).

#### Data analysis and software

All data preprocessing, statistical analysis, machine-learning modeling, model evaluation, and figure generation were performed using R (v4.5.2) in a local computational environment. Core packages included ggplot2, corrplot, caret, randomForest, e1071, xgboost, lightgbm, and other supporting libraries for data manipulation and visualization. Exact software versions are listed in the key resources table.

### Quantification and statistical analysis

Model performance was quantified using the R^2^, RMSE, and MAE. All metrics were computed under resampling-based validation with preprocessing steps fully nested within each fold.

Where applicable, agreement between categorical ecological risk tiers was summarized using overall accuracy and Cohen’s κ. Detailed statistical outputs, sample sizes, and performance summaries are reported in the results and discussion section, figure legends, and Supplementary Tables.

#### Additional resources

Not applicable.
