# Supplementary material for: Machine learning-driven prediction of heavy metal pollution for aquatic health surveillance and disease control
Source: iScience. 2026 Jul 7;29(7):116549. doi: 10.1016/j.isci.2026.116549 (PMC13378350; doi:10.1016/j.isci.2026.116549)
Supplement: Document S1. Tables S1–S4 and Appendix A [file mmc1.pdf]

**iScience, Volume *29***

## Supplemental information

# Machine learning-driven prediction of heavy metal pollution for aquatic health surveillance and disease control

**Md. Abdullah Al Mamun Hridoy, Pakorn Ditthakit, Akib Hosen, Mohd. Saifur Rahman, Minhaj Uddin, Kamrul Hasan, Yeaman Hossain, Chiara Bordin, Leonardo Goliatt, Md. Abdullah Al Mamun, and Paolo Pastorino**

Supplementary Information. Supplementary Tables S1–S4 provide (i) confusion matrices and misclassification summaries for PLI/RI/Zn-Igeo, (ii) Zn hotspot sensitivity (measured vs predicted), and (iii) bootstrap-based uncertainty propagation and class-stability diagnostics.

**Table S1.** Confusion matrices comparing measured vs predicted risk-tier classifications for PLI, RI, and Zn-Igeo.

**S1a:** PLI confusion matrix

| **Measured \ Predicted** | **0 (PLI < 1)** | **1 (PLI ≥ 1)** |
|---|---|---|
| 0 (PLI < 1) | 20 | 3 |
| 1 (PLI ≥ 1) | 3 | 4 |

**S1b:** RI confusion matrix (Hakanson)

| **Measured \ Predicted** | **0 (RI < 150)** |
|---|---|
| 0 (RI < 150) | 28 |
| 1 (150–300) | 2 |

**S1c:** Zn-Igeo confusion matrix

| Measured \ Predicted | 0 (≤0) | 1 (0–1) |
|---|---|---|
| 0 (≤0) | 24 | 2 |
| 1 (0–1) | 2 | 1 |
| 2 (1–2) | 1 | 0 |

**Table S2.** Zn hotspot sensitivity: measured vs predicted Zn and resulting Zn-Igeo class shifts. Metals in mg $kg^{-1}$; Igeo is dimensionless.

| Sample | Zn (mg/kg) | Zn_pred (mg/kg) | Igeo_Zn | Igeo_Zn_pred | Igeo class | Pred class |
|---|---|---|---|---|---|---|
| R7 | 295.681 | 66.146 | 1.557 | -0.603 | 2 | 0 |
| R4 | 118.276 | 48.675 | 0.235 | -1.046 | 1 | 0 |
| R9 | 103.243 | 139.121 | 0.039 | 0.469 | 1 | 1 |
| R8 | 102.828 | 59.275 | 0.033 | -0.762 | 1 | 0 |
| R6 | 90.506 | 110.094 | -0.151 | 0.132 | 0 | 1 |
| T4 | 79.961 | 25.740 | -0.330 | -1.965 | 0 | 0 |
| R13 | 68.209 | 137.585 | -0.559 | 0.453 | 0 | 1 |
| R12 | 65.538 | 85.387 | -0.617 | -0.235 | 0 | 0 |

**Table S3.** Samples with lowest Zn-Igeo class stability (bootstrap), recommended for confirmatory sampling.

| Sample | Zn | Zn_pred | Igeo_Zn | Igeo_Zn_pred | Igeo class | Pred class | Stability |
|---|---|---|---|---|---|---|---|
| R6 | 90.51 | 110.09 | -0.151 | 0.132 | 0 | 1 | 0.472 |

| | | | | | | | |
|---|---|---|---|---|---|---|---|
| R5 | 48.19 | 94.31 | -1.060 | -0.092 | 0 | 0 | 0.738 |
| R1 | 50.97 | 93.50 | -0.980 | -0.104 | 0 | 0 | 0.777 |
| R9 | 103.24 | 139.12 | 0.039 | 0.469 | 1 | 1 | 0.790 |
| R13 | 68.21 | 137.58 | -0.559 | 0.453 | 0 | 1 | 0.798 |

**Table S4.** Coefficient of determination ($R^2$) for each machine learning model across target metals

| | As | Cd | Cu | Hg | Ni | Pb | Zn |
|---|---|---|---|---|---|---|---|
| **LightGBM** | NA | NA | NA | NA | NA | NA | NA |
| **AdaBoost** | NA | NA | NA | NA | NA | NA | NA |
| **SVM** | 0.15 | 0.94 | 0.78 | 0.1 | 0.92 | 0.73 | 0.74 |
| **KNN** | NA | 0.95 | 1 | NA | 0.22 | 0.89 | 0.86 |
| **RF** | 0.15 | 0.68 | 0.79 | 0.44 | NA | 0.72 | 0.92 |
| **ANN** | 0.41 | 0.3 | 0.49 | 0.82 | 0.26 | 0.37 | 0.9 |
| **XGB** | 0.04 | 0.9 | 0.83 | NA | 0.23 | 0.93 | 0.38 |

**Appendix Information**

Appendix A (Tables A1–A2) provides complete hyperparameter tuning ranges and final model configurations used in this study.

**Appendix A**

**Table A1.** Hyperparameter search spaces and tuning design for each algorithm (search ranges, search strategy, and cross-validation settings).

| Algorithm | Hyperparameters tuned | Search space (range/list) | Search strategy | Inner CV | Selection criterion |
|---|---|---|---|---|---|
| Random Forest (RF) | n_estimators; max_depth; min_samples_split; | n_estimators: [300, 500, 800, 1200]; max_depth: [None, 5, 10, 20]; min_samples_split: [2, 5, | Random / Grid | 5-fold | Min RMSE; |

| Algorithm | Hyperparameters tuned | Search space (range/list) | Search strategy | Inner CV | Selection criterion |
|---|---|---|---|---|---|
| | min_samples_leaf; max_features | 10]; min_samples_leaf: [1, 2, 4]; max_features: [sqrt, 0.5, 1.0] | | | MAE as tie-breaker |
| KNN | n_neighbors; weights; p | n_neighbors: [3, 5, 7, 9, 11]; weights: [uniform, distance]; p: [1, 2] | Grid | 5-fold | Min RMSE; MAE tie-breaker |
| SVR | kernel; C; gamma; epsilon | kernel: [rbf, linear]; C: [1, 10, 50, 100]; gamma: [scale, 0.01, 0.1, 1]; epsilon: [0.01, 0.05, 0.1] | Grid / Random | 5-fold | Min RMSE |
| XGBoost | n_estimators; learning_rate; max_depth; subsample; colsample_bytree; reg_lambda | n_estimators: [200–2000]; learning_rate: [0.01–0.3]; max_depth: [2–8]; subsample: [0.6–1.0]; colsample_bytree: [0.6–1.0]; reg_lambda: [0–10] | Random | 5-fold | Min RMSE |
| LightGBM | num_leaves; learning_rate; n_estimators; min_child_samples; subsample; colsample_bytree | num_leaves: [15–127]; learning_rate: [0.01–0.3]; n_estimators: [200–2000]; min_child_samples: [5–50]; subsample: [0.6–1.0]; colsample_bytree: [0.6–1.0] | Random | 5-fold | Min RMSE |
| ANN | hidden_layer_sizes; alpha; learning_rate_init; max_iter; early_stopping | hidden_layer_sizes: [(20,), (50,), (30,30), (50,50)]; alpha: [1e−5–1e−2]; learning_rate_init: [1e−4–1e−2]; max_iter: [2000–8000]; early_stopping: [True] | Random | 5-fold | Min RMSE |
| AdaBoost | n_estimators; learning_rate; base_estimator_depth | n_estimators: [100–1500]; learning_rate: [0.01–1.0]; depth: [1–5] | Random | 5-fold | Min RMSE |

**Table A2.** Final selected hyperparameters and cross-validated performance summaries for each metal model combination.

| Target metal | Final model | Final hyperparameters (selected) | CV protocol | $R^2$ (mean ± SD) | RMSE (mean ± SD) | MAE (mean ± SD) |
|---|---|---|---|---|---|---|
| As | RF | n_estimators = …; max_depth = …; min_samples_leaf = …; max_features = … | Outer 5-fold (nested) | … ± … | … ± … | … ± … |
| Cd | KNN | n_neighbors = …; weights = …; p = … | Outer 5-fold (nested) | … ± … | … ± … | … ± … |
| Cu | KNN | n_neighbors = …; weights = …; p = … | Outer 5-fold (nested) | … ± … | … ± … | … ± … |
| Hg | ANN | hidden_layer_sizes = …; alpha = …; learning_rate_init = …; early_stopping = True | Outer 5-fold (nested) | … ± … | … ± … | … ± … |
| Ni | SVR | kernel = rbf; C = …; gamma = …; epsilon = … | Outer 5-fold (nested) | … ± … | … ± … | … ± … |
| Pb | XGBoost | n_estimators = …; learning_rate = …; max_depth = …; subsample = …; colsample_bytree = …; reg_lambda = … | Outer 5-fold (nested) | … ± … | … ± … | … ± … |
| Zn | RF | n_estimators = …; max_depth = …; min_samples_leaf = …; max_features = … | Outer 5-fold (nested) | … ± … | … ± … | … ± … |
